# Supplementary material for: Molecular basis for PICS-mediated piRNA biogenesis and cell division
Source: Nat Commun. 2021 Sep 22;12:5595. doi: 10.1038/s41467-021-25896-7 (PMC8458385; doi:10.1038/s41467-021-25896-7)
Supplement: Supplementary file 1 — Supplementary Information [file 41467_2021_25896_MOESM1_ESM.pdf]

Supplementary Information

Molecular basis for PICS-mediated piRNA biogenesis and cell division

Xiaoyang Wang[1,#], Chenming Zeng[1,#], Shanhui Liao[1,#], Zhongliang Zhu[1], Jiahai Zhang[1], Xiaoming Tu[1], Xuebiao Yao[1], Xuezhu Feng[1,*], Shouhong Guang[1,2,*], Chao Xu[1,*]

[1] Ministry of Education Key Laboratory for Membraneless Organelles & Cellular Dynamics, Hefei National Laboratory for Physical Sciences at the Microscale, School of Life Sciences, Division of Life Sciences and Medicine, University of Science and Technology of China

[2]Department of Obstetrics and Gynecology, The First Affiliated Hospital of USTC, Division of Life Sciences and Medicine, University of Science and Technology of China, 230027, Hefei, P.R. China

[#]These authors contributed equally: Xiaoyang Wang, Chenming Zeng, and Shanhui Liao

*To whom correspondence should be addressed. E-mail: fengxz@ustc.edu.cn; sguang@ustc.edu.cn; xuchaor@ustc.edu.cn.

Supplementary Table 1. ITC binding affinities for PICS subunits and their variants

Proteins or peptides (syringe)	Proteins (cell)	Molecular Ratio (N)	(K_d: μM)
PICS-1^{RRM}	TOFU-6^{RRM}	1.1	0.0062 ± 0.0009
F217E(PICS-1^{RRM})	TOFU-6^{RRM}	0.7	0.57 ± 0.07
K246A/F247A(PICS-1^{RRM})	TOFU-6^{RRM}	1.1	17 ± 4
Y250A/Q251A(PICS-1^{RRM})	TOFU-6^{RRM}	1.2	21 ± 0.5
PICS-1^{RRM}	D26A/W27A(TOFU-6^{RRM})	0.7	30 ± 5.0
SUMO	ERH-2	N/A	*NB
SUMO-PICS-$1^{180\text{-}200}$	ERH-2	0.7	8.5 ± 2.0
I182D/V186D(SUMO-PICS-$1^{180\text{-}200}$)	ERH-2	N/A	NB
V189D/L190D(SUMO-PICS-$1^{180\text{-}200}$)	ERH-2	N/A	NB
SUMO-PICS-$1^{180\text{-}200}$	D67A(ERH-2)	0.8	4.3 ± 0.7
SUMO-PICS-$1^{180\text{-}200}$	M38D(ERH-2)	N/A	NB
SUMO-TOST-$1^{34\text{-}54}$	ERH-2	0.9	3.2 ± 0.5
SUMO-TOST-$1^{34\text{-}54}$	D67A(ERH-2)	N/A	NB
SUMO-TOST-$1^{34\text{-}54}$	ERH-2-$(GSS)_7$-PICS-$1^{180\text{-}200}$	0.9	4.9 ± 1.7
R42C(SUMO-TOST-$1^{34\text{-}54}$)	ERH-2	N/A	NB
L39A/F43A(SUMO-TOST-$1^{34\text{-}54}$)	ERH-2	N/A	NB
SUMO-PID-$1^{50\text{-}70}$	ERH-2	1.0	18 ± 2.0
SUMO-PID-$1^{50\text{-}70}$	ERH-2-$(GSS)_7$-PICS-$1^{180\text{-}200}$	1.3	12 ± 2.0
SUMO-PID-$1^{50\text{-}70}$	ERH-2-$(GSS)_6$-TOST-$1^{34\text{-}54}$	N/A	NB
SUMO-PID-$1^{50\text{-}70}$	D67A(ERH-2)	N/A	NB

Dissociation constants were measured using isothermal titration calorimetry (ITC). Dissociation constants (K_ds) were from a minimum of two experiments (mean ± SD).

*NB: No detectable binding.

Source data for ITC binding are provided as a Source Data file.

Supplementary Table 2. **Primers used for RT and qRT assays**

Primer name	Sequence (5' to 3')
21UR-1 RT primer	GTTGGCTCTGGTGCAGGGTCCGAGGTATTCGCACCAGAGCCAACGCACGG
21UR-3442 RT primer	GTTGGCTCTGGTGCAGGGTCCGAGGTATTCGCACCAGAGCCAACCACAATCTC
21UR-5045 RT primer	GTTGGCTCTGGTGCAGGGTCCGAGGTATTCGCACCAGAGCCAACGCCGTT
21UR-1 qRT primer F	GCCGTGGTACGTACGTTAAC
21UR-3442 qRT primer F	GCGGCGGTACTAGAGTGTT
21UR-5045 qRT primer F	GCCCGTGCCAAACTCCATTTA
piRNA qRT universal primer R	GTGCAGGGTCCGAGGTATT

Supplementary Table 3. Primers used for mutations

Primers	Sequences (5' to 3')
PICS-1 F217E	GGCATGCTGAACACCGAAGGTATTGCGCAACTG<br>CAGTTGCGCAATACCTTCGGTGTTCAGCATGCC
PICS-1 K246A/F247A	GTTGCGCTGGAGAACGCGGCGCAGGTGTATCAAGCG<br>CGCTTGATACACCTGCGCCGCGTTCTCCAGCGCAAC
PICS-1 Y250A/Q251A	AACAAGTTCCAGGTGGCGGCGGCGGTTCAGGACTTT<br>AAAGTCCTGAACCGCCGCCGCCACCTGGAACTTGTT
PICS1 V189D/L190D	CATTAAGGATAGCGTGTTCAAAGATGATCATGCGGAGGAAGAGCCGCGTG<br>CACGCGGCTCTTCCTCCGCATGATCATCTTTGAACACGCTATCCTTAATG
PICS-1 I182DV186D	ATTCCATATGGAAGACATTAAGGATAGCGTGTTCAAAGTTCTGCATG<br>CATGCAGAACTTTGAACACGCTATCCTTAATGTCTTCCATATGGAAT
TOFU-6 D26A/W27A	CCGAAAGCGTGGAACGCTGCGAACCTGTTCCACGTT<br>AACGTGGAACAGGTTCGCAGCGTTCCACGCTTTCGG
TOST-1 R42C	ATCACATTGAATGAGTGCTTCGGAGTTCTCGAA<br>TTCGAGAACTCCGAAGCACTCATTCAATGTGAT
TOST-1 L39A/F43A	AATAAACGAATCACAGCGAATGAGCGCGCGGGAGTTCTCGAAAAG<br>CTTTTCGAGAACTCCCGCGCGCTCATTCGCTGTGATTCGTTTATT
ERH-2 D67A	CTCGATAAGCTCTCCGCGGTTTCTATGATGATC<br>GATCATCATAGAAACCGCGGAGAGCTTATCGAG

Supplementary Table 4. Strains used in this work

Strains	Genotypes
N2	N2
OD139	ItIs37[pie-1p::mcherry::his58 IV;pals3502[pie-1::YFP::LMN-1]
TM2417	pics-1(tm2417/hT2) I
SHG357	ustIS32[TOFU-6::GFP] II
SHG514	ustIS32[TOFU-6::GFP] II;pics-1(tm2417/hT2) I
SHG538	ustIS58[TOST-1::GFP] II
SHG542	ustIS60[PICS-1::GFP] II
SHG583	ustIS60[PICS-1::GFP] II;pics-1(tm2417) I
SHG654	ustIS73[ERH-2::GFP] III in situ
SHG772	ustIS82[PICS-1::GFP] V
SHG670	ustIS55[PID-1::GFP] II
SHG777	tofu-6(ust94/mIn1) II
SHG784	ustIS83[TOFU-6::GFP] V
SHG794	ustIS82[PICS-1::GFP] V;tofu-6(ust94/mIn1) II
SHG798	ustIS77[ERH-2::GFP] III;tofu-6(ust94/mIn1)
SHG803	ustIS83[TOFU-6::GFP] V; tofu-6(ust94) II
SHG809	tofu-6(ust94/mIn1) II; pkIS3 [Ppie-1::GFP::H2B] I
SHG829	erh-2(ust101/qC1) III
SHG831	tost-1(ust103/qC1) III
SHG835	ustIS58[TOST-1::GFP] II;tost-1(ust103) III
SHG851	ustIS60[PICS-1::GFP] II;erh-2(ust101/qC1) III
SHG1002	tofu-6(ust173) II
SHG1008	ustIS111[PICS-1(K245A/F246A)::GFP] II
SHG1014	ustIS118[PICS-1(Y250A/Q251A)::GFP] II
SHG1021	ustIS111[PICS-1(K245A/F246A)::GFP] II;pics-1(tm2417) I
SHG1024	ustIS82[PICS-1::GFP] V;*tofu-6(ust173)*
SHG1025	ustIS77[ERH-2::GFP] III;tofu-6(ust173)
SHG1027	ustIS121[PICS-1(F217E)::GFP] II
SHG1028	tofu-6(ust173) II;pkIS3 [Ppie-1::GFP::H2B] I
SHG1029	ustIS118[PICS-1(Y250A/Q251A)::GFP] II;pics-1(tm2417) I
SHG1034	pics-1(tm2417/hT2) I;ItIs37[pie-1p::mcherry::his58] IV
SHG1043	ustIS143[TOFU-6(D26AW27A)::GFP] V
SHG1052	ustIS121[PICS-1(F217E)::GFP] II;pics-1(tm2417) I
SHG1053	ustIS143[TOFU-6(D26AW27A)::GFP] V; tofu-6(ust94) II
SHG1091	ustIS118[PICS-1(Y250A/Q251A)::GFP] II;pics-1(tm2417) I;ustIS144[LMN-1::mCherry] I
SHG1180	ustIS111[PICS-1(K245A/F246A)::GFP] II;pics-1(tm2417) I;ItIs37[pie-1p::mcherry::his58] IV
SHG1187	ustIS121[PICS-1(F217E)::GFP] II;pics-1(tm2417) I;ItIs37[pie-1p::mcherry::his58] IV
SHG1188	ustIS60[PICS-1::GFP] II;pics-1(tm2417) I;ItIs37[pie-1p::mcherry::his58] IV
SHG1433	ustIS171[TOST-1(L39A/F43A)::GFP] II
SHG1435	ustIS173[TOST-1(R42C)::GFP II

SHG1474	ustIS178[PICS-1(I182D/V186D)::GFP II
SHG1475	ustIS179[PICS-1(V189D/L190D)::GFP II
SHG1481	ustIS182[ERH-2::GFP] II
SHG1482	ustIS183[ERH-2(D67A)::GFP] II
SHG1484	ustIS171[TOST-1(L39A/F43A)::GFP] II;tost-1(ust103/qC1) III
SHG1486	ustIS173[TOST-1(R42C)::GFP] II;tost-1(ust103/qC1) III
SHG1488	ustIS182[ERH-2::GFP] II;erh-2(ust101) III
SHG1498	ustIS118[PICS-1(Y250A/Q251A)::GFP] II;pics-1(tm2417) I;ItIs37[pie-1p::mcherry::his58] IV
SHG1499	ustIS183[ERH-2(D67A)::GFP] II;erh-2(ust101/qC1) III
SHG1508	ustIS178[PICS-1(I182D/V186D)::GFP] II;pics-1(tm2417/hT2) I
SHG1509	ustIS178[PICS-1(I182D/V186D)::GFP] II;pics-1(tm2417/hT2) I;ItIs37[pie-1p::mcherry::his58] IV
SHG1510	ustIS179[PICS-1(V189D/L190D)::GFP] II;pics-1(tm2417/hT2) I
SHG1511	ustIS179[PICS-1(V189D/L190D)::GFP] II;pics-1(tm2417/hT2) I;ItIs37[pie-1p::mcherry::his58] IV
SHG1512	tost-1(ust103/qC1) III;ItIs37[pie-1p::mcherry::his58] IV
SHG1514	erh-2(ust101/qC1) III;ItIs37[pie-1p::mcherry::his58] IV
SHG1515	ustIS182[ERH-2::GFP] II;tost-1(ust103/qC1) III
SHG1516	ustIS58[TOST-1::GFP] II;erh-2(ust101/qC1) III
SHG1521	tost-1(ust103) III;ItIs37[pie-1p::mcherry::his58] IV
SHG1522	ustIS58[TOST-1::GFP] II;tost-1(ust103) III;ItIs37[pie-1p::mcherry::his58] IV
SHG1523	ustIS171[TOST-1(L39A/F43A)::GFP] II;tost-1(ust103/qC1) III;ItIs37[pie-1p::mcherry::his58] IV
SHG1524	ustIS172[TOST-1(L39D/F43D)::GFP] II;tost-1(ust103/qC1) III;ItIs37[pie-1p::mcherry::his58] IV
SHG1525	ustIS173[TOST-1(R42C)::GFP] II;tost-1(ust103/qC1) III;ItIs37[pie-1p::mcherry::his58] IV
SHG1526	ustIS182[ERH-2::GFP] II;pics-1(tm2417/hT2) I
SHG1527	ustIS189[PID-1(R61C):GFP] V
SHG1528	ustIS182[ERH-2::GFP] II;erh-2(ust101) III;ItIs37[pie-1p::mcherry::his58] IV
SHG1529	ustIS183[ERH-2(D67A)::GFP] II;erh-2(ust101/qC1) III;ItIs37[pie-1p::mcherry::his58] IV
SHG1540	ustIS55[PID-1::GFP] II; erh-2(ust101/qC1)
SHG1546	ustIS190[ERH-2(M38D)::GFP] II
SHG1578	ustIS190[ERH-2(M38D)::GFP] II;erh-2(ust101/qC1)
SHG1612	ustIS190[ERH-2(M38D)::GFP] II;erh-2(ust101/qC1);ItIs37[pie-1p::mcherry::his58] IV
YY513	pkIS32[Ppie-1::GFP::H2B] I

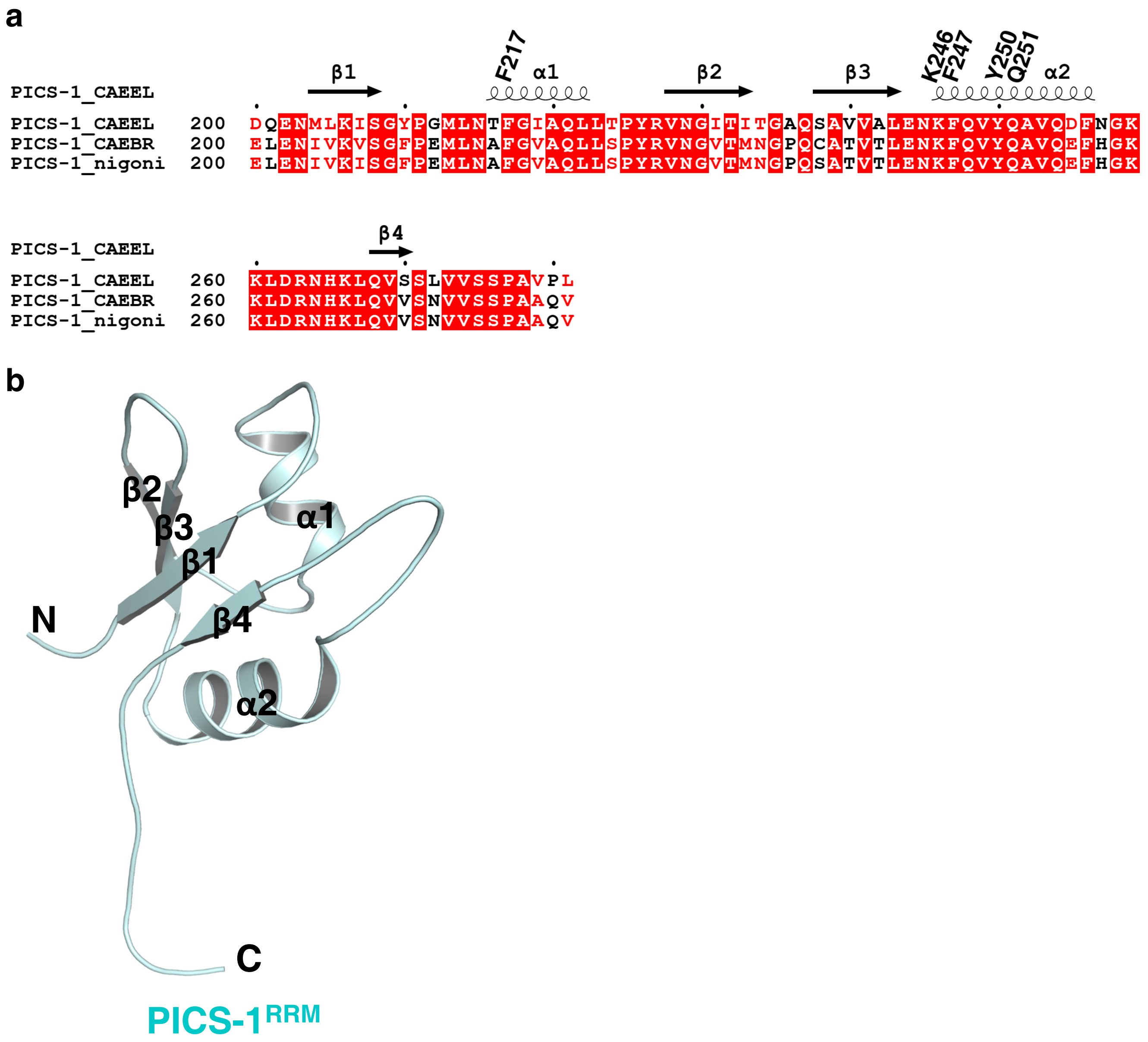

Supplementary Fig. 1. The structure of PICS-1^{RRM}. a Sequence alignement of the RRM domain of PICS-1 orthologs from *Caenorhabditis elegans* (PICS-1_CAEEL, NP_491011.1) , *Caenorhabditis briggsaeand* (PICS-1_CAEBR, XP_002639160.1), and *Caenorhabditis nigoni* (PICS-1_nigoni, PIC55854.1). The secondary strucures of *Caenorhabditis elegans* PICS-1^{RRM} are labelled at the top of the sequences. **b** Structure of the RRM domain of PICS-1 is shown in cyan cartoon, with secordary structure labelled.

Supplementary Fig. 1

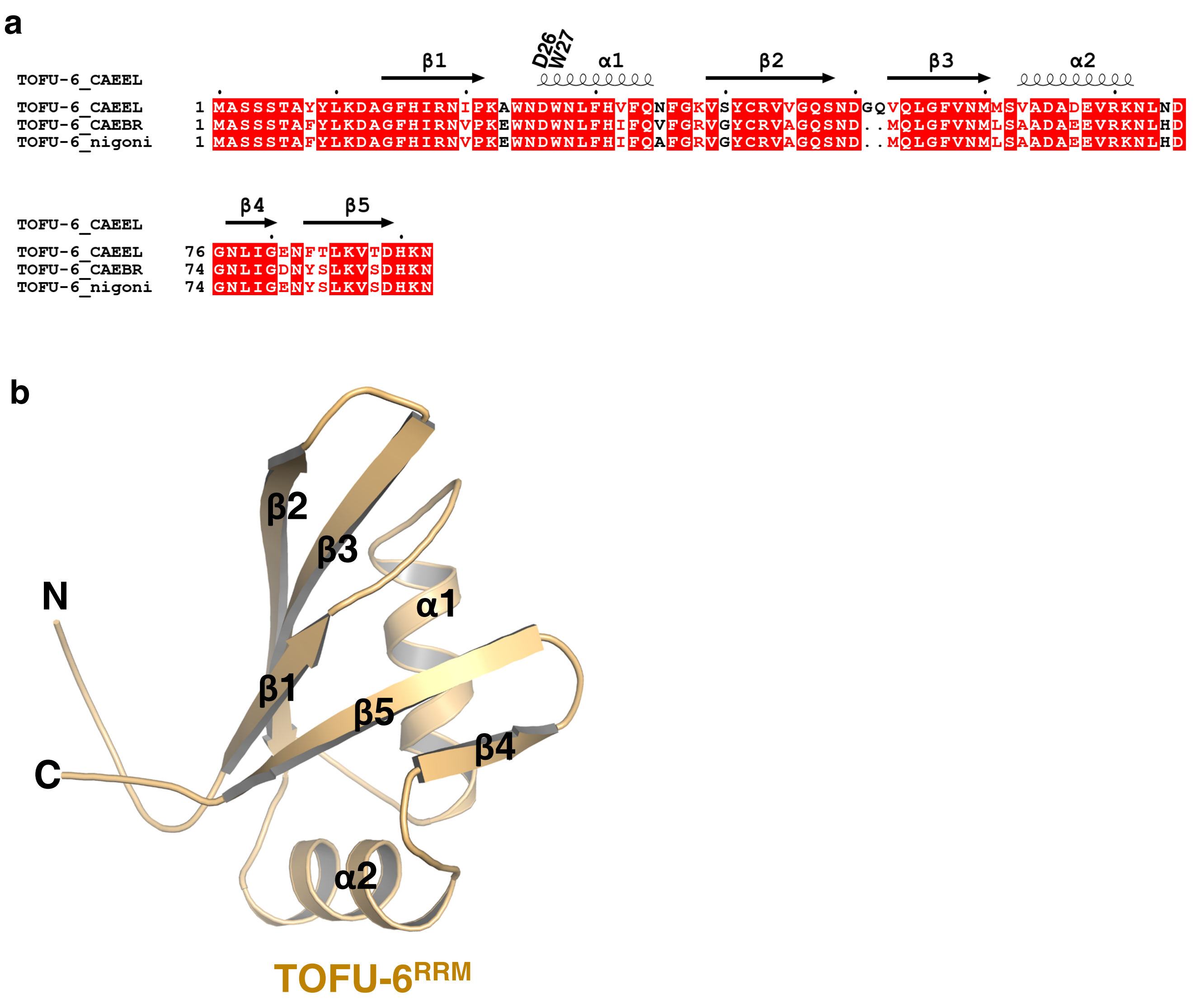

Supplementary Fig. 2. The structure of TOFU-6^{RRM}. a Sequence alignement of the RRM domain of TOFU-6 orthologs from *Caenorhabditis elegans* (TOFU-6_CAEEL, NP_001293507.1), *Caenorhabditis briggsaeand* (TOFU-6_CAEBR, XP_002630711.1), and *Caenorhabditis nigoni* (TOFU-6_nigoni, PIC46813.1).). The secondary strucures of *Caenorhabditis elegans* TOFU-6^{RRM} are labelled at the top of the sequences. **b** Structure of the RRM domain of TOFU-6 is shown in orange cartoon, with secordary structure labelled.

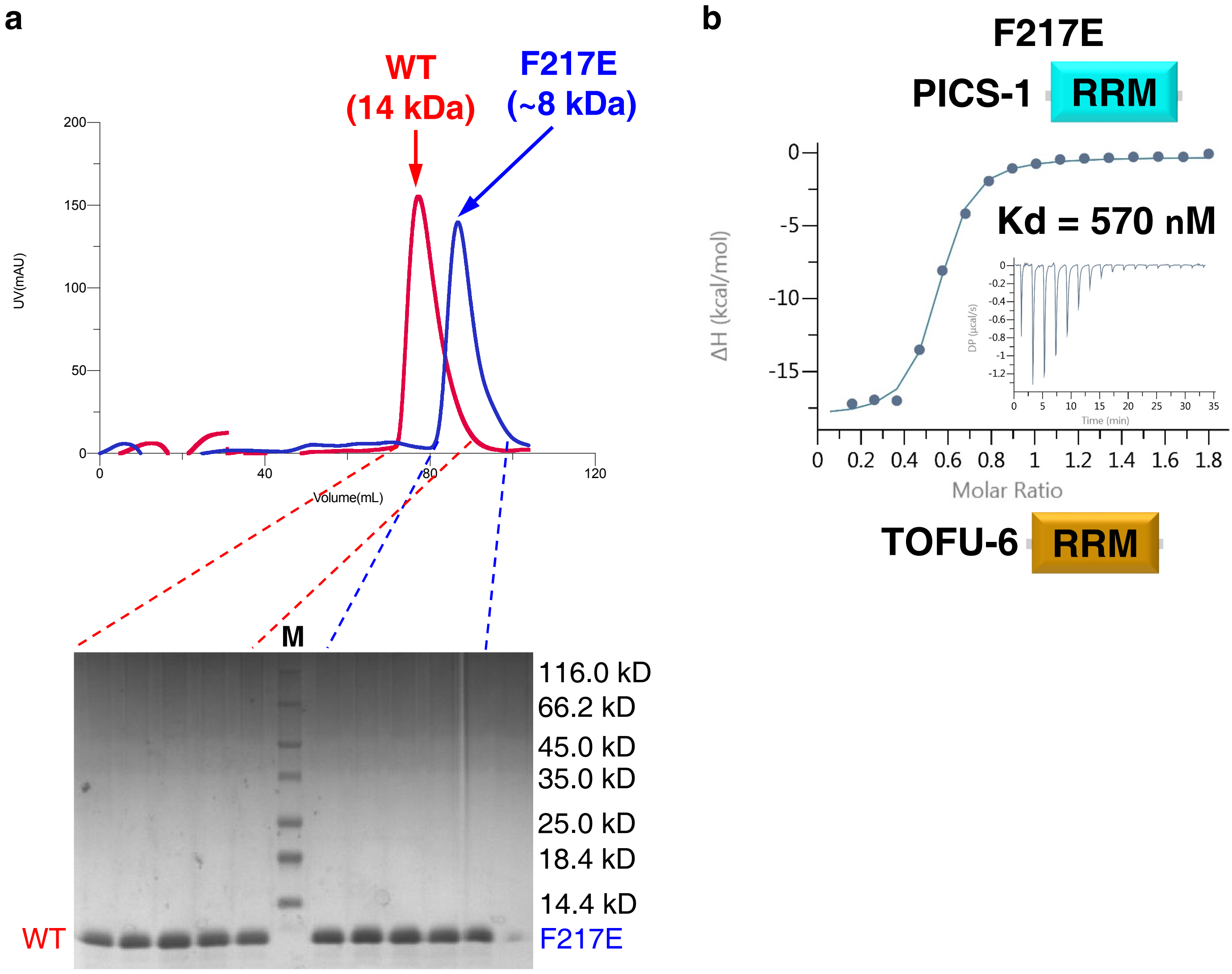

Supplementary Fig. 3. F217E disrupts the PICS-1^{RRM} homodimer. a Gel-filtration chromatography of wild type and F217E of PICS-1^{RRM}, indicating the molecular weights of ~14kD and 8kD, respectively (upper panel). The SDS-PAGE gel of wild type and F217E of PICS-1^{RRM}, with the Marker in the middle (lower panel). The gel filtration buffere is 20 mM Tris, pH7.5, 200 mM NaCl. **b** ITC binding curve for the binding of PICS-1^{RRM} F217E to TOFU-6^{RRM}. The gel filtration experiments were peformed twice independently. Source data for gel image are provided as a Source Data file.

Supplementary Fig. 3

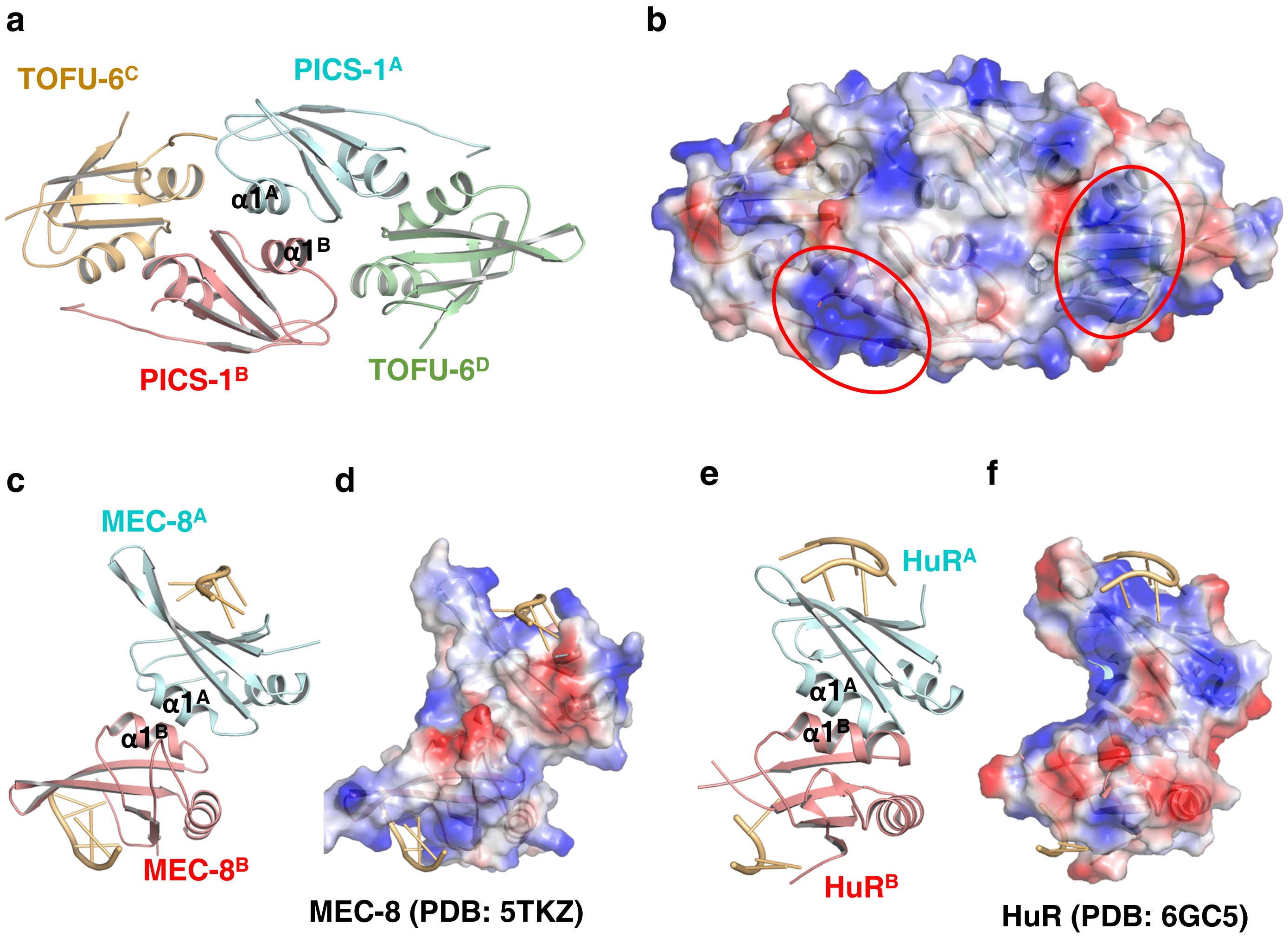

Supplementary Fig. 4. The structure of homodimerized RRM domain. a Overall structure of PICS-1^{RRM}-TOFU6^{RRM} tetramer. The color code is the same as shown in Fig. 1c. **b** Electrostatic surface of the PICS-1^{RRM}-TOFU6^{RRM} tetramer, with potential RNA binding sites indicated by red circles. **c** Overall structure of the MEC-8 RRM homodimer (PDB: 5TKZ). The two promers shown in cyan and red cartoon, respectively, and the RNAs are shown in oragen cartoon. **d** Electrostatic surface of the MEC-8 RRM homodimer. **e** Overall structure of the HuR RRM homodimer (PDB: 6GC5). The two promers shown in cyan and red cartoon, respectively, and the RNAs are shown in oragen cartoon. **f** Electrostatic surface of the HuR RRM homodimer.

Supplementary Fig. 4

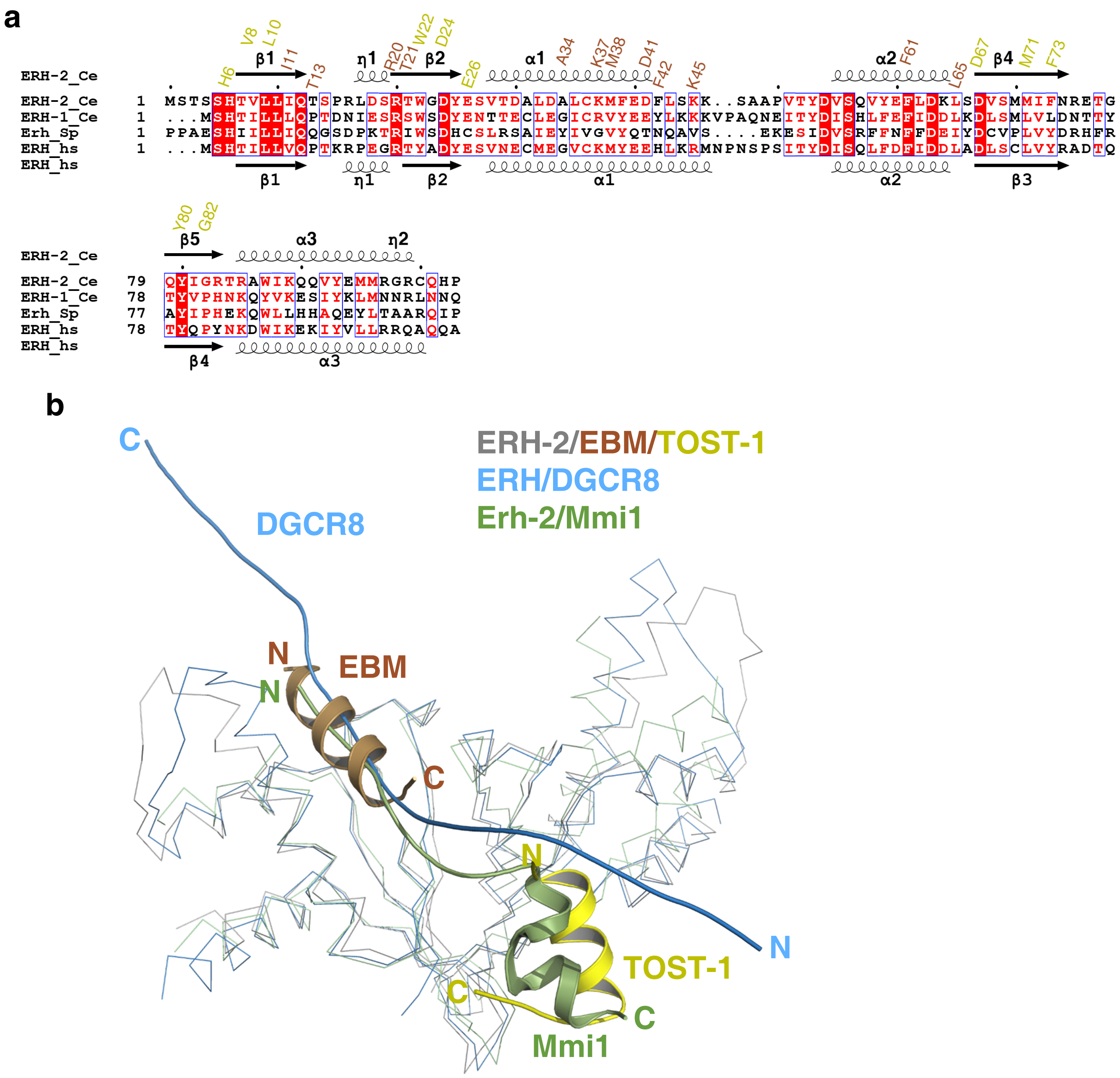

Supplementary Fig. 5. Comparison of the structures of ERH orthologs. a Sequence alignment of ERH-2 orthologs, including ERH-2 (ERH-2_Ce, NP_497936.2) and ERH-1 (ERH-1_Ce, NP_505713.1) of *Caenorhabditis elegans,* Erh1 of *Schizosaccharomyces pombe* (Erh_Sp, XP_004001791.1)*,* and *homo sapiens* ERH (ERH_hs, NP_004441.1). Secondary structures of *Caenorhabditis elegans* ERH-2 and *homo sapiens* ERH are labelled at the top and bottom of sequences, respectively. The ERH-2 residues involved in binding to EBM and TOST-1 are highlighted in brown and yellow, respectively. **b** Structural superposition of ERH-2(gray)/EBM(brown) and ERH-2(gray)/TOST-1(yellow) of *Caenorhabditis elegans*, human ERH/DGCR8 (blue), and *Schizosaccharomyces pombe* Erh-2/Mmi1 (green). ERH orthologs and ERH-binding ligands are shown in ribbon and in cartoon, respectively.

Supplementary Fig. 5

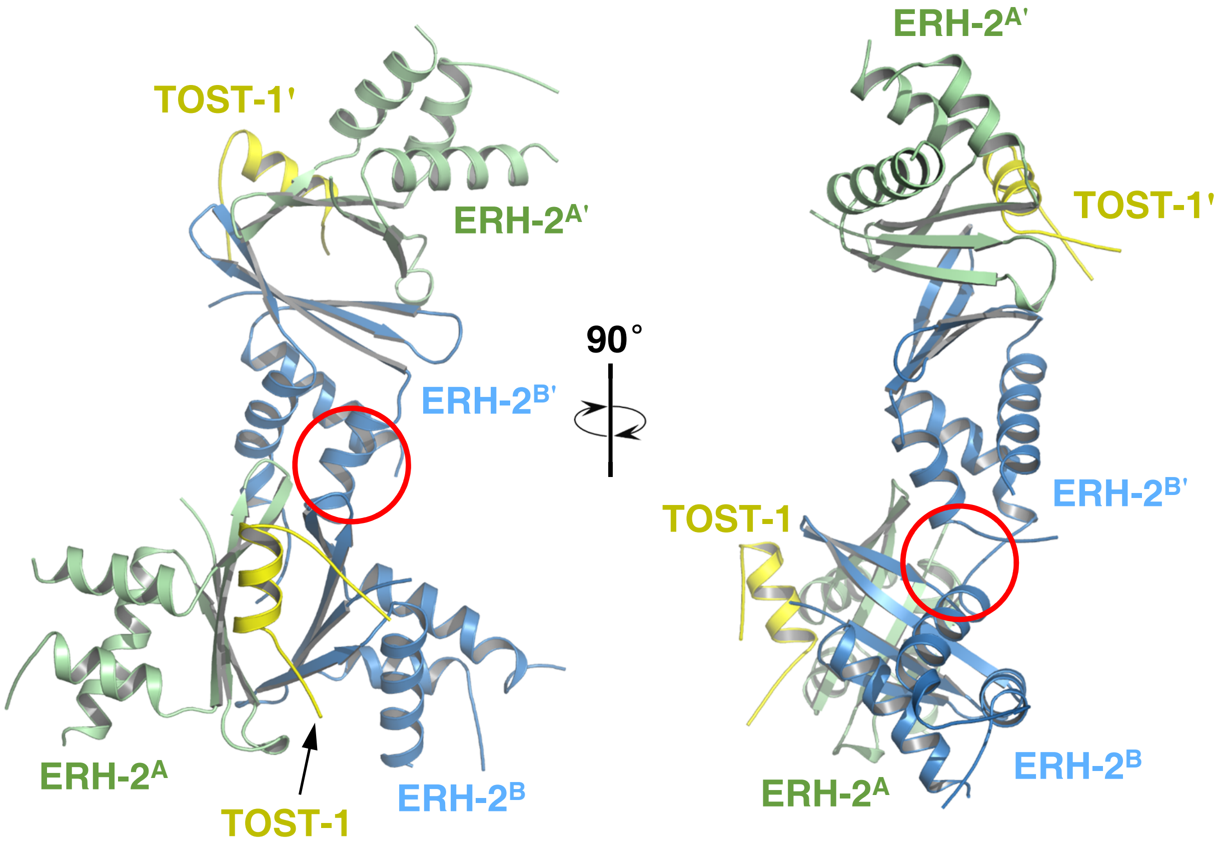

Supplementary Fig. 6. Crystal packing blocks the binding of TOST-1 to ther other site of the ERH-2 dimer. In the two adjacent ERH-2/TOST-1 complexes, ERH-2^A, ERH-2^B, and TOST-1 are shown in green, blue, and green cartoon, respectively. Only one TOST-1 molecular is visible. Due to crystal packing, potential binding site for the other TOST-1 is blocked by one ERH-2 protomer (ERH-$2^{B'}$) from the adjacent ERH-2 dimer .

Supplementary Fig. 6

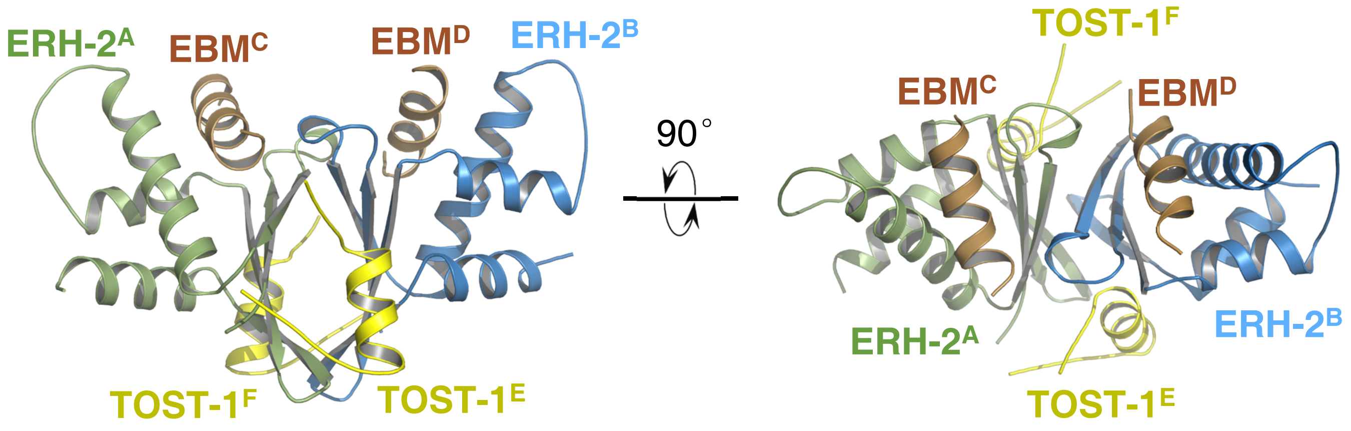

Supplementary Fig. 7. ERH-2 is capable of binding to PICS-1^{EBM} and TOST-1 simultaneously. Based on the structures of ERH-2/EBM and ERH-2/TOST-1, Two TOST-1 molecules (TOST-1^{E} and TOST-1^{F}) are shown in yellown cartoon and modelled into the ERH-2/EBM complex, which is shown in the same way as in Fig. 3b. The two EBM and two TOST-1 molecules bind to different suffaces of ERH-2 dimer.

Supplementary Fig. 7

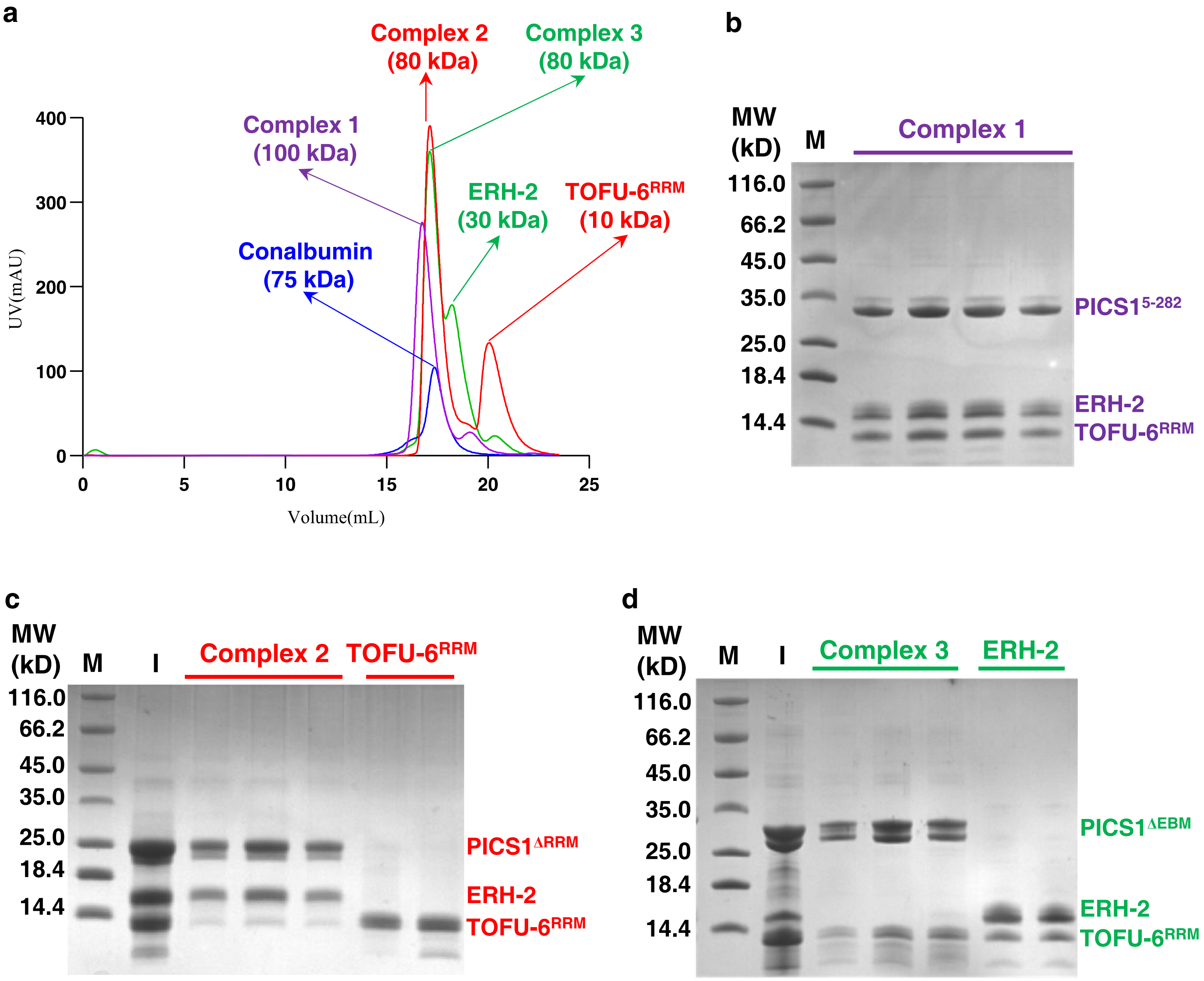

Supplementary Fig. 8. Gel filtration experiments for PICS subcomplexes. a gel filtration profiles for different PICS subcomplexes with their peaks indicated by arrows of different colors. PICS1$^{\Delta EBM}$: PICS-1$^{5\text{-}282}$ with EBM deleted. PICS1$^{\Delta RRM}$: PICS-1$^{5\text{-}200}$. Complex 1: TOFU-6^{RRM}/PICS-1$^{5\text{-}282}$/ERH-2; Complex 2: PICS1$^{\Delta RRM}$/ERH-2; Complex 3: TOFU-6^{RRM}/PICS1$^{\Delta EBM}$. Conalbumin (75 kD) is used as the marker. **b** The SDS-PAGE for the peaks of the complex 1, indicates that the three proteins form a complex in a ratio of 2:2:2. M: marker; I: input. **c** The SDS-PAGE for the peaks of the complex 2, indicates that PICS1$^{\Delta RRM}$/ERH-2 no longer binds to TOFU-6^{RRM}. **d** The SDS-PAGE for the peaks of the complex 3, indicates that TOFU-6^{RRM}/PICS1$^{\Delta EBM}$ no longer binds to ERH-2. The gel filtration experiments were peformed twice independently. Source data for gel images are provided as a Source Data file

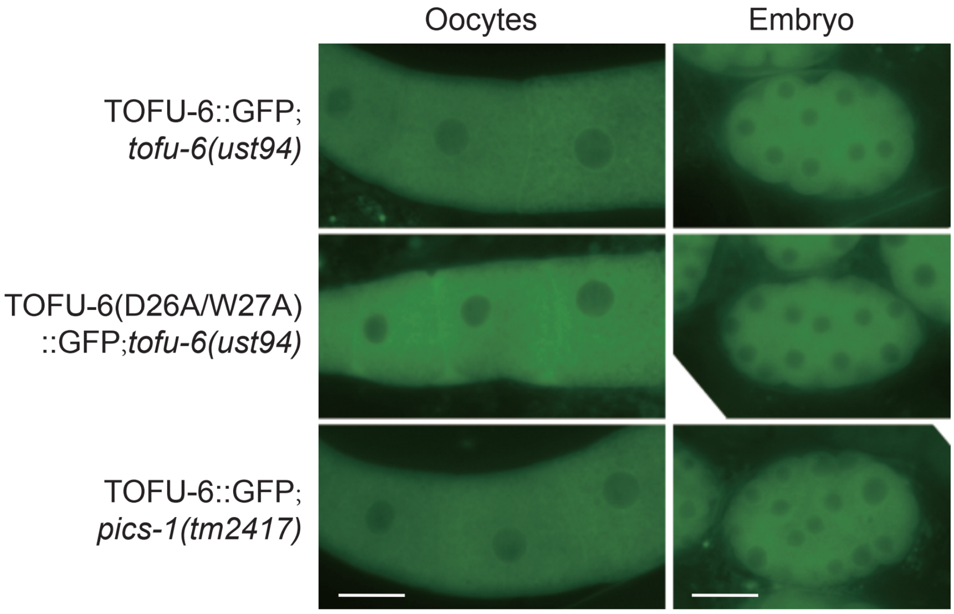

Supplementary Fig. 9. The expressions TOFU-6 in oocytes and embryos in indicated mutant backgrounds. Images of oocytes and embryos expressing GFP tagged-TOFU-6 or TOFU-6(D26A/W27A) in indicated mutant backgrounds. TOFU-6(D26A/W27A) disrupts the interaction between TOFU-6 and PICS-1. Scale bar, 20μm. The worms were harvested once, while the imaging experiments were triplicated.

Supplementary Fig. 9

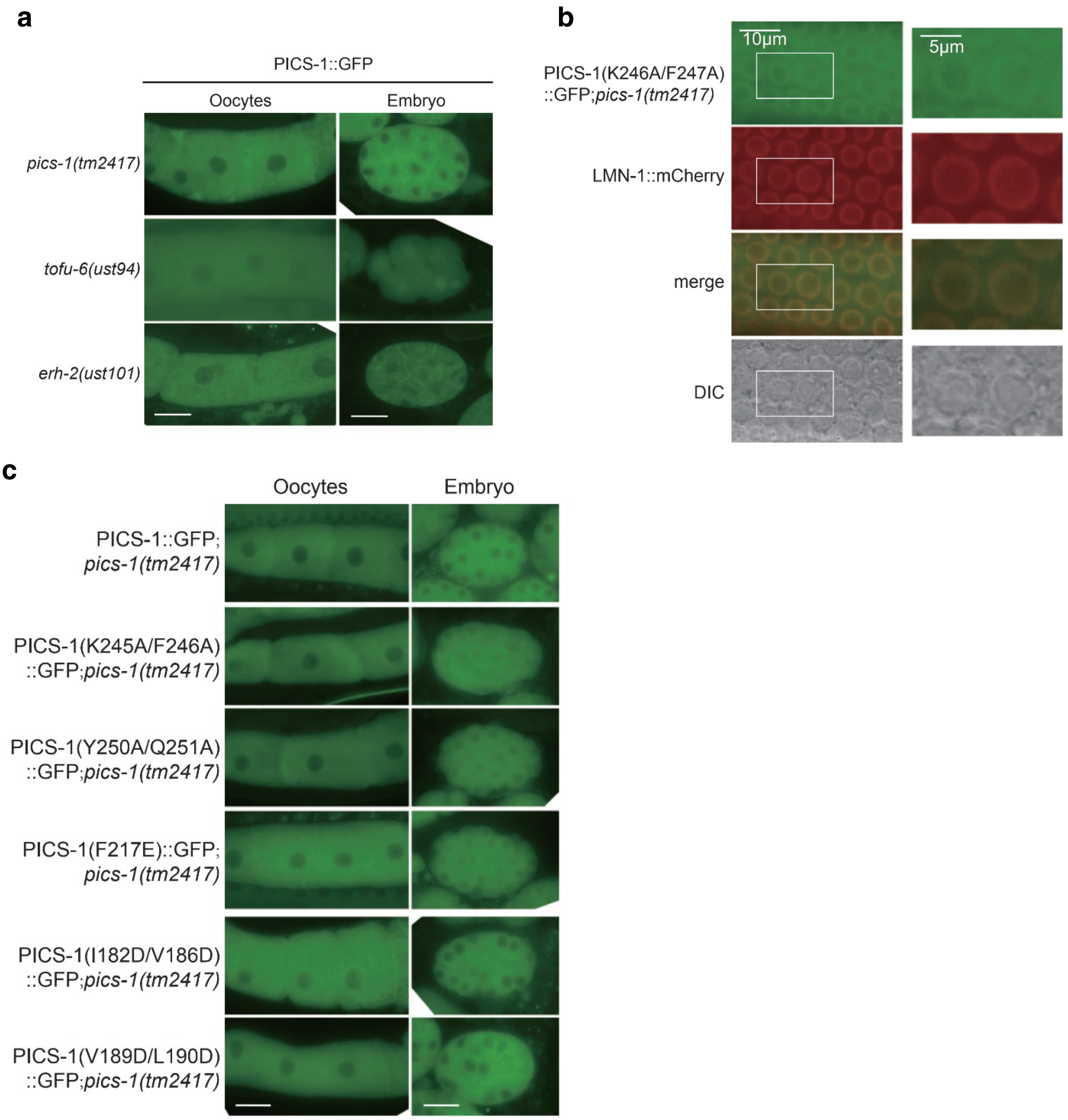

Supplementary Fig. 10. The expressions of PICS-1 in oocytes and embryos in indicated mutant backgrounds. a Images of oocytes and embryos expressing GFP tagged PICS-1 in indicated mutant backgrounds. Scale bar, 20μm. **b** Images of PICS-1(K246A/F247A)::GFP;pics-1(tm2417) and nuclear membrane marker LMN-1::mCherry in adult germ cells. **c** Images of oocytes and embryos expressing GFP tagged wild-type or amino acids- substituted PICS-1 in indicated mutant backgrounds. PICS-1(K245A/F246A) and PICS-1(Y250A/Q251A) disrupt the interaction between TOFU-6 and PICS-1. PICS-1(F217E) disrupts the dimerization of PICS-1. PICS-1(I182D/V186D) and PICS-1(V189D/L190D) disrupt the interaction between PICS-1 and ERH-2. Scale bar, 20μm. The worms were harvested once, while the imaging experiments were triplicated.

Supplementary Fig. 10

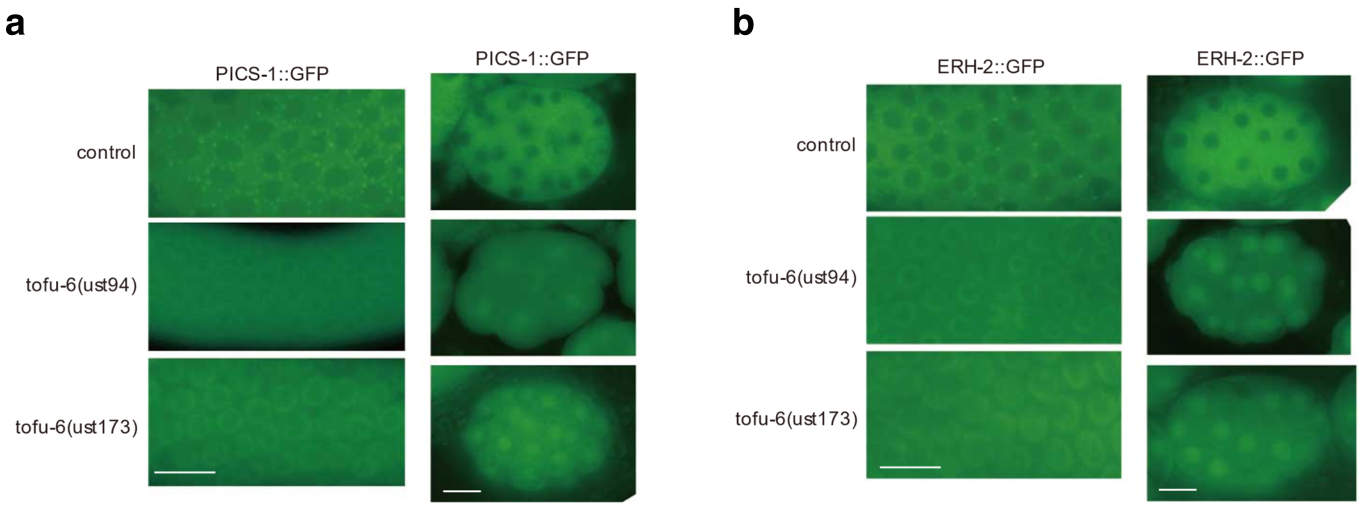

Supplementary Fig. 11. The expressions of PICS-1 and ERH-2 are dependent on the interactions of PICS complexes. **a** Images of adult germline cells (left) and embryos (right) expressing GFP tagged- PICS-1 in *tofu-6* mutant backgrounds. **b** Images of adult germline cells (left) and embryos (right) expressing GFP tagged- ERH-2 in *tofu-6* mutant backgrounds. Scale bar, 20µm. The worms were harvested once, while the imaging experiments were triplicated.

Supplementary Fig. 11

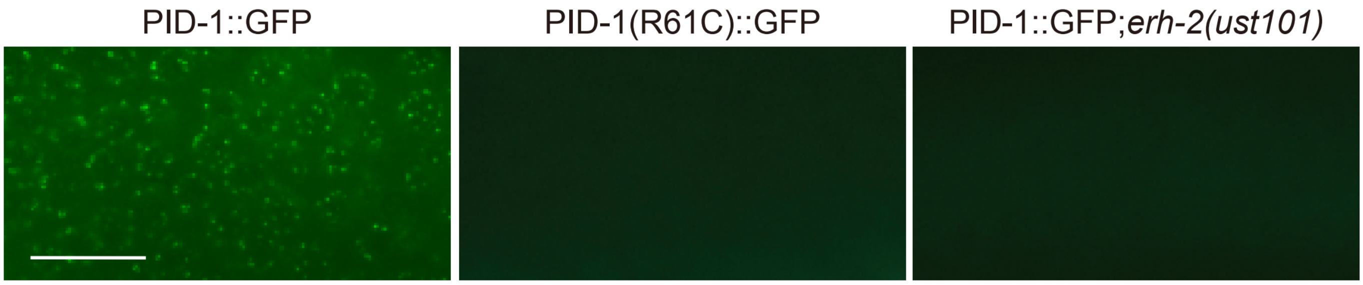

Supplementary Fig. 12. The expressions of PID-1 in indicated mutant backgrounds. Images of germ-cells expressing GFP-tagged-PID-1 or PID-1(R61C) and GFP-tagged-PID-1 in erh-2(ust101) mutant background. PID-1(R61C) disrupts the interaction between PID-1 and ERH-2. Scale bar, 20µm. The worms were harvested once, while the imaging experiments were triplicated.

Supplementary Fig. 12

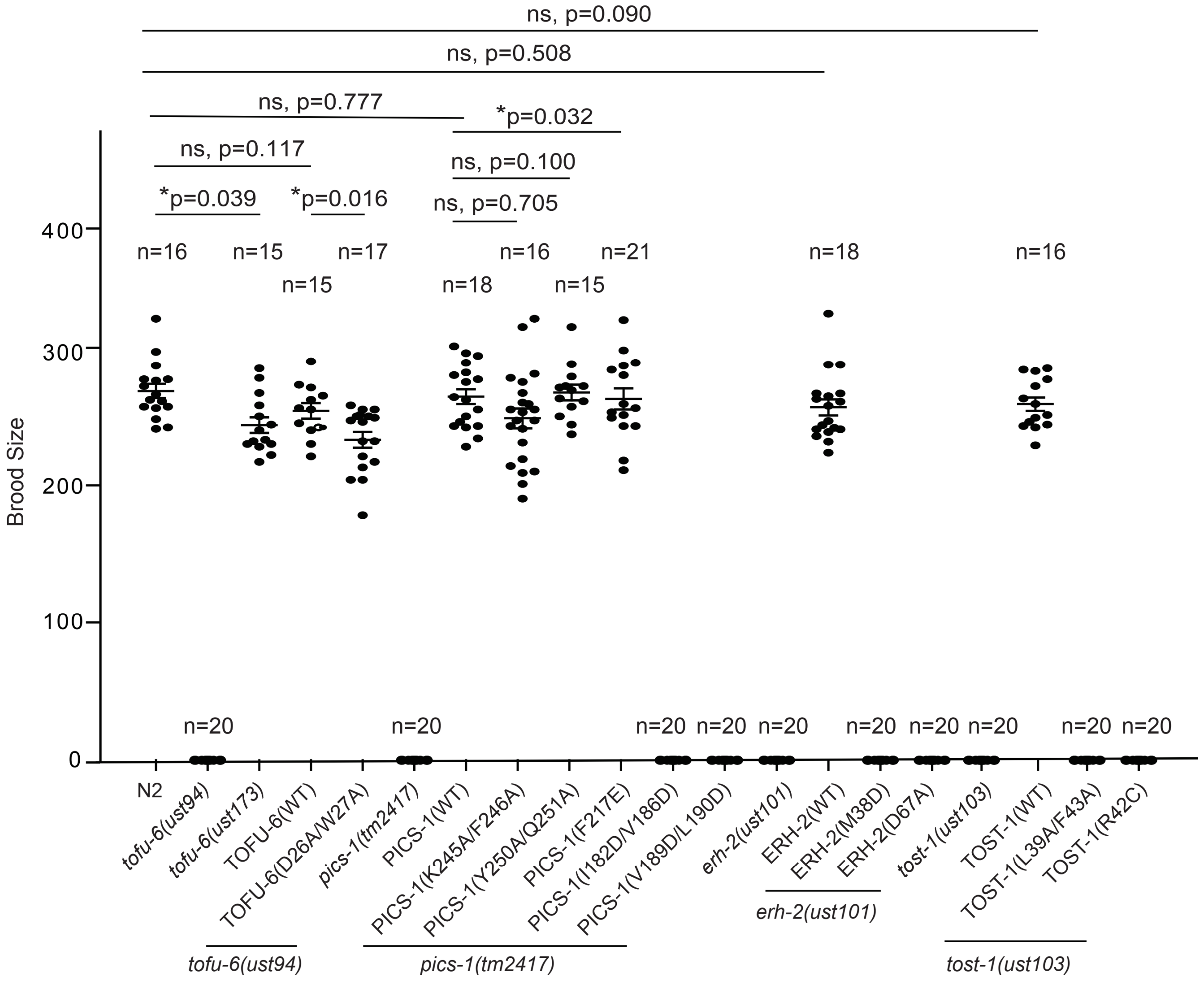

Supplementary Fig. 13. Disrupting PICS-1/ERH-2 and ERH-2/TOST-1 interactions, but not TOFU-6/PICS-1 interaction, leads to embryonic lethality. Bar graph displaying the brood sizes in indicated worms. Worms were grown at 20℃, mean ± SD; *p<0.05; ns, not significant; n= 15, 16, 17, 18, 20 or 21; two-tailed paired t-tests.

Supplementary Fig. 13

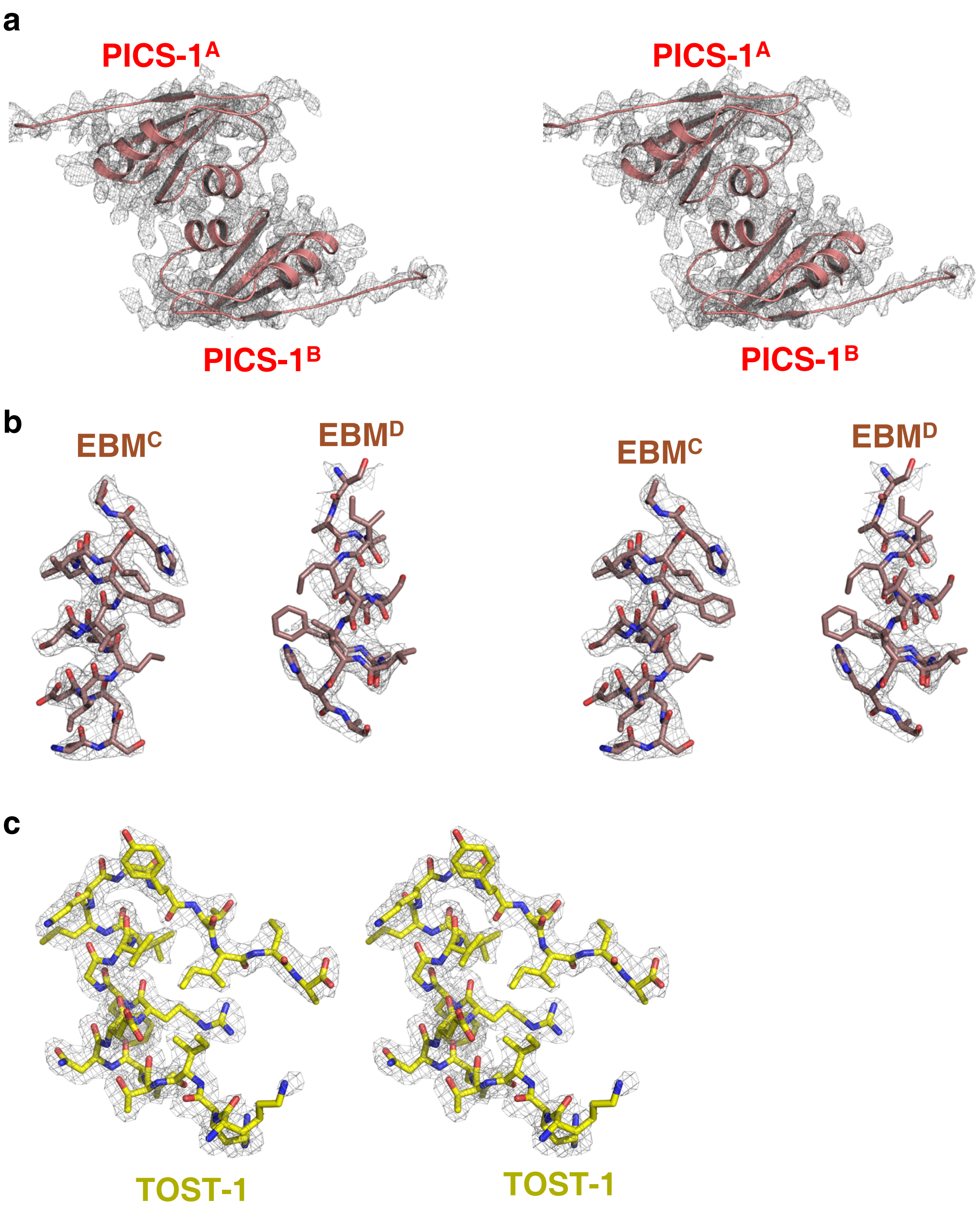

Supplementary Fig. 14. Stereo images of representative 2|Fo|–|Fc| maps for crystal structures contoured at 1.0 σ. a The PICS-1^{RRM} homodimer; **b** Two EBM molecules in the ERH-2/EBM complex; **c** The TOST-1$^{34\text{-}54}$ molecule in the ERH-2/TOST-1 complex.
